# Sarcosine Remodels DNA Methylation-Linked Transcriptional Networks During Epileptogenesis in the Rat Rapid Hippocampal Kindling Model

**DOI:** 10.3390/ijms27177938

**Published:** 2026-09-06

**Authors:** Nicole Ferris, Lan Phung, Wakaba Omi, Guoku Hu, Hai-Ying Shen

**Affiliations:** 1 Division of Neurology, Department of Pediatrics, University of Nebraska Medical Center, Children’s Nebraska, Omaha, NE 68198, USA; 2 Child Health Research Institute, University of Nebraska Medical Center, Children’s Nebraska, Omaha, NE 68198, USA; 3 Department of Translational Neuroscience, Legacy Research Institute, Portland, OR 97232, USA; 4 Department of Pharmacology & Experimental Neuroscience, University of Nebraska Medical Center, Omaha, NE 68198, USA

**Keywords:** DNA methylation, epileptogenesis, sarcosine, GlyT1 inhibition, rapid hippocampal kindling, differentially methylated regions (DMRs), epigenetic regulation, hippocampus

## Abstract

DNA methylation is implicated in epileptogenesis. Sarcosine, a glycine transporter 1 (GlyT1) inhibitor and methyl donor, attenuates behavioral progression during rapid hippocampal kindling and alters hippocampal DNA methylation, but its locus-specific epigenetic effects remain poorly understood. Here, reduced representation bisulfite sequencing (RRBS) was combined with targeted gene expression analysis in the hippocampi of sarcosine-treated kindled rats. RRBS identified 563, 533, and 390 differentially methylated regions (DMRs), corresponding to 521, 499, and 374 DMR-associated genes, in vehicle-kindled versus sham (vKD vs. vSH), sarcosine-kindled versus sham (sKD vs. vSH), and sarcosine-kindled versus vehicle-kindled (sKD vs. vKD) comparisons, respectively. Pathway enrichment analysis identified 217 significantly affected pathways, including glutamatergic signaling, extracellular matrix (ECM) organization, chromatin regulation, axon guidance, and apoptotic processes. Eleven candidate genes involved in epigenetic regulation, excitatory neurotransmission, and ECM remodeling were selected for transcriptional validation. All 11 genes were significantly upregulated in kindled hippocampi, whereas sarcosine was associated with reduced expression relative to vehicle-kindled rats for eight genes (*Hdac9*, *Fos*, *Smad7*, *Unc5a*, *Grik2*, *Gpr37l1*, *Cacna2d2*, and *Yy1*). Collectively, these findings indicate that sarcosine remodels DNA methylation-associated transcriptional networks during rapid hippocampal kindling and support GlyT1 inhibition as a potential disease-modifying approach in experimental epileptogenesis.

## 1. Introduction

Epileptogenesis refers to the progressive transformation of a functionally normal brain into an epileptic state, culminating in the emergence of spontaneous, recurrent seizures. This pathological process is often initiated by neurological insults, such as traumatic brain injury, stroke, central nervous system infection, or status epilepticus [1,2]. Although antiseizure medications (ASMs) are effective in suppressing acute and chronic seizures, they do not prevent or reverse the underlying development of epilepsy [3]. These limitations underscore a significant unmet need for disease-modifying therapies that can intervene during the latent period and halt progression to chronic epilepsy. Here, we investigate how sarcosine treatment, which includes GlyT1 inhibition among its biological actions, is associated with locus-specific DNA methylation and transcriptional changes during epileptogenesis in a rat rapid hippocampal kindling model.

Genome-wide DNA methylation alterations, often accompanied by increased DNA methyltransferase (DNMT) activity, have been reported in patients with temporal lobe epilepsy (TLE), pediatric epilepsy, and multiple experimental epilepsy models [4,5,6,7,8,9]. These findings suggest that epigenetic dysregulation is associated with diverse forms of epilepsy [3,4,10,11,12,13]. Importantly, DNA methylation changes are highly context-dependent, with distinct epilepsy models exhibiting unique methylation signatures [14,15]. Both hypermethylation and hypomethylation occur during epileptogenesis and affect a wide range of genomic regions, including CpG islands, promoters, gene bodies, and intergenic loci [10,13]. Because the functional effects of DNA methylation depend on genomic location, cell type, chromatin context, and the specific gene involved, methylation changes cannot be assumed to predict a uniform direction of transcriptional change. These epigenetic alterations are therefore best viewed as components of broader activity-dependent molecular remodeling that may be associated with dysregulation of gene networks involved in inflammation, synaptic remodeling, neuroplasticity, and neuronal survival.

DNA methylation changes during epileptogenesis are also highly cell-type specific, differentially affecting neuronal and glial populations [3,12]. Several epilepsy-associated genes exhibit coordinated changes in DNA methylation and gene expression, suggesting a potential regulatory relationship, although the connection between methylation status and transcriptional output is often complex and context-dependent [3,7]. Importantly, co-occurrence of a DMR and altered expression does not establish a direct regulatory or causal relationship, particularly when DMRs are assigned to genes by genomic proximity. Despite increasing recognition of the role of epigenetic mechanisms in epilepsy, no currently available therapy has been shown to halt or reverse the epigenetic processes that may contribute to epileptogenesis. While certain ASMs, such as valproic acid, influence DNA methylation and histone acetylation, their impact on long-term disease progression remains limited [16]. Likewise, non-pharmacological interventions such as the ketogenic diet can partially modify methylation profiles in experimental models but have not consistently prevented epileptogenesis in at-risk populations [11,17]. Identifying methylation-associated genes and pathways that change during epileptogenesis, and determining whether these alterations can be therapeutically reversed, remain important challenges in the development of disease-modifying therapies [3,18].

We previously demonstrated that sarcosine (N-methylglycine), a GlyT1 inhibitor and methyl donor, attenuated behavioral progression during epileptogenesis in a rat kindling model [19]. In addition to inhibiting glycine reuptake, sarcosine participates in one-carbon metabolism and may influence cellular methylation potential [20]. Sarcosine may also influence glutamatergic and glycinergic signaling, including indirect effects on NMDA receptor activity; therefore, the present study cannot attribute its molecular effects exclusively to GlyT1 inhibition, methyl-donor activity, or one-carbon metabolism. Our prior work further showed that sarcosine decreases hippocampal 5-methylcytosine (5mC) levels while increasing 5-hydroxymethylcytosine (5hmC), a pattern consistent with altered DNA methylation/demethylation dynamics during epileptogenesis [19]. These findings motivated examination of methylation-associated molecular networks but do not establish that DNA methylation changes mediate sarcosine’s behavioral effects.

To further define the epigenetic mechanisms underlying sarcosine’s effects in rapid hippocampal kindling, we employed our established hippocampal kindling model and performed reduced representation bisulfite sequencing (RRBS) together with targeted gene expression analysis of hippocampal tissue from sham, kindled, and sarcosine-treated animals. Our objectives were to identify gene- and pathway-level methylation changes associated with rapid hippocampal kindling and determine whether sarcosine treatment was associated with altered epigenetic profiles. We hypothesized that kindling-induced DNA methylation changes would be associated with network remodeling and that sarcosine treatment would be associated with altered methylation-linked transcriptional networks involved in neuronal excitability, synaptic plasticity, and structural remodeling. While previous studies have largely cataloged methylation changes associated with epilepsy, few have directly linked genome-wide methylation remodeling to a candidate disease-modifying intervention and corresponding transcriptional changes. By integrating RRBS, pathway enrichment analyses, and targeted gene expression profiling, the present study characterizes DNA methylation-linked and transcriptional networks associated with sarcosine treatment during rapid hippocampal kindling.

## 2. Results

### 2.1. Sarcosine Attenuates Seizure Progression During Rapid Hippocampal Kindling

**Figure 1:**
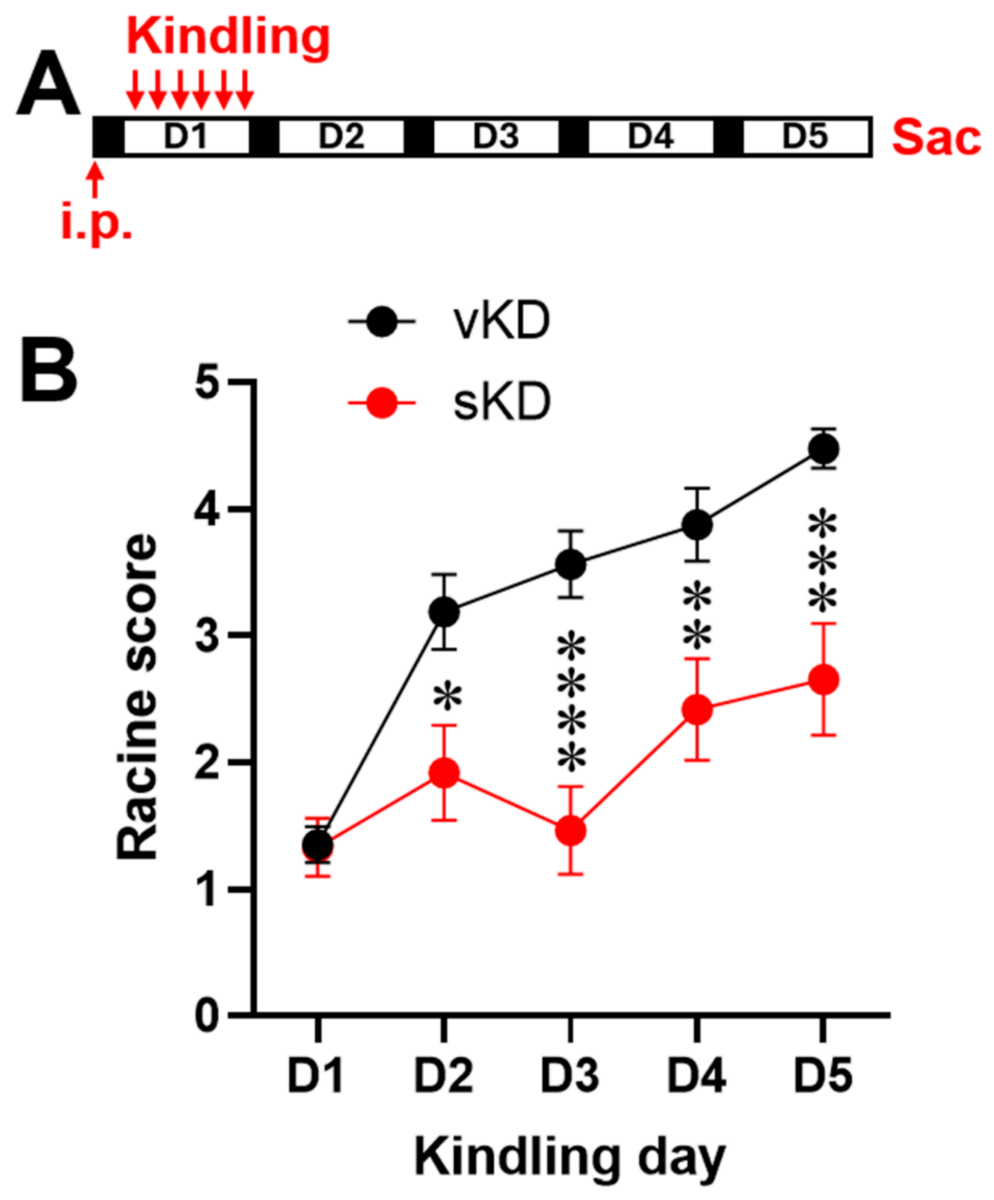
**Sarcosine attenuates behavioral seizure progression during rapid hippocampal kindling.** (**A**) Experimental timeline. Vehicle or sarcosine (Sac; 3 g/kg, i.p.) was administered 30 min before each daily kindling session. The red upward arrow indicates i.p. administration, and the red downward arrows indicate kindling stimulations. Hippocampi were collected 24 h after the final stimulation. (**B**) Racine seizure scores in vehicle-kindled (vKD) and sarcosine-kindled (sKD) rats across 5 days of kindling. Data are mean ± SEM (n = 6 per group). * *p* < 0.05, ** *p* < 0.01, *** *p* < 0.001, **** *p* < 0.0001 vs. vKD at the corresponding time point.

To confirm the previously observed effect of sarcosine on behavioral kindling progression in the cohort used for methylation analyses, rats underwent the previously established rapid hippocampal kindling protocol (Figure 1A) [19]. Vehicle-treated kindled rats (vKD) developed fully kindled behavioral seizures over five days of stimulation, reaching Racine stage 4–5 seizures by the first stimulation on day 5. Their mean Racine score on day 5 was 4.6 ± 0.3 (mean ± SEM; n = 6, Figure 1B).

In contrast, sarcosine-treated rats (sKD; 3 g/kg, i.p., administered daily) displayed lower behavioral seizure severity, with a mean Racine score of 2.5 ± 0.8 on day 5 (n = 6, *p* < 0.05 versus vKD; Figure 1B). Thus, under this rapid hippocampal kindling protocol, sarcosine treatment was associated with attenuated behavioral seizure progression in the animals subsequently used for methylation and gene-expression analyses, consistent with our previous findings [19].

### 2.2. Genome-Wide DNA Methylation Changes Associated with Kindling and Sarcosine Treatment

**Figure 2:**
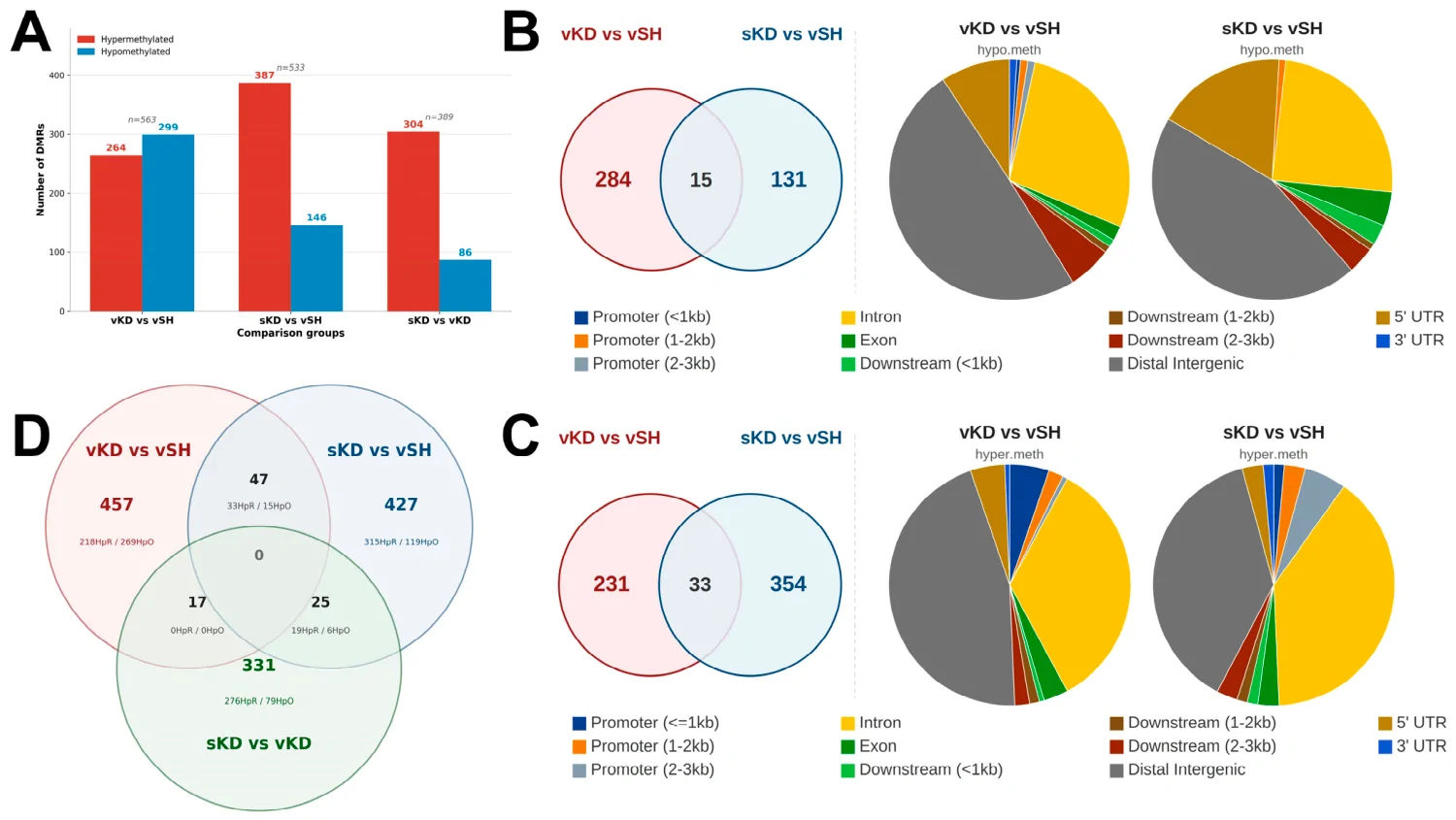
**Hippocampal DMR profiles in kindled and sarcosine-treated rats.** (**A**) Numbers of hypermethylated and hypomethylated DMRs in the vKD vs. vSH, sKD vs. vSH, and sKD vs. vKD comparisons (|Δmethylation| ≥ 10%, q < 0.05). (**B**,**C**) Overlap and genomic distribution relative to transcription start sites (TSSs) of hypomethylated (**B**) and hypermethylated (**C**) DMRs in the vKD vs. vSH and sKD vs. vSH comparisons. (**D**) Overlap of DMR-associated genes across the three comparisons. Candidate genes selected for qPCR were derived from sarcosine-relevant DMR-associated gene sets and pathway-enrichment analyses. See Supplementary Table S4 for DMR annotations and functional categories of qPCR candidates.

To characterize hippocampal DNA methylation patterns associated with kindling-induced epileptogenesis and sarcosine treatment, we identified genome-wide differentially methylated regions (DMRs) across experimental groups. Compared with non-kindled sham controls (vSH), vehicle-treated kindled rats (vKD) exhibited 264 hypermethylated and 299 hypomethylated DMRs (|Δmethylation| ≥ 10%, q < 0.05), indicating widespread differences in hippocampal methylation profiles during kindling (Figure 2A). Sarcosine-treated kindled rats (sKD) exhibited 387 hypermethylated and 146 hypomethylated DMRs relative to vSH, with 33 hypermethylated and 15 hypomethylated DMRs shared between vKD vs. vSH and sKD vs. vSH contrasts, indicating a subset of methylation changes common to both kindled groups. In the sKD vs. vKD contrast, 304 hypermethylated and 86 hypomethylated DMRs were identified, such that more DMRs showed higher methylation in sKD than in vKD (Figure 2A). To characterize shared and distinct methylation signatures, overlap analyses were performed separately for hypomethylated and hypermethylated DMRs. Among hypomethylated DMRs, 284 were unique to vKD vs. vSH, 131 were unique to sKD vs. vSH, and 15 were shared between the two contrasts (Figure 2B, left panel). Among hypermethylated DMRs, 231 were unique to vKD vs. vSH, 354 were unique to sKD vs. vSH, and 33 were shared (Figure 2C, left panel). The genomic distributions of hypo- and hypermethylated DMRs relative to transcription start sites are shown in Figure 2B and Figure 2C, respectively. Notably, sarcosine-treated animals exhibited a greater proportion of hypermethylated DMRs located within the 1–3 kb upstream interval relative to transcription start sites than vehicle-treated kindled animals. This positional distribution does not, by itself, establish an effect on transcription.

DMRs were subsequently assigned to their nearest transcription start sites (TSSs) to generate DMR-associated gene sets for downstream pathway and transcriptional analyses. These assignments indicate genomic proximity and do not demonstrate direct methylation–expression relationships. Across all contrasts, 563, 533, and 390 DMRs mapped to 521, 499, and 374 unique DMR-associated genes in the vKD vs. vSH, sKD vs. vSH, and sKD vs. vKD groups, respectively (Figure 2D). Venn analysis identified three pairwise overlap gene sets: 47 genes shared between vKD vs. vSH and sKD vs. vSH, 17 genes shared between vKD vs. vSH and sKD vs. vKD, and 25 genes shared between sKD vs. vSH and sKD vs. vKD. No genes were shared across all three contrasts. The 25 DMR-associated genes shared between the sKD vs. vSH and sKD vs. vKD contrasts were designated a shared sarcosine-associated DMR-gene set, because DMRs assigned to these genes were detected in both sham-referenced and kindling-referenced comparisons. This designation reflects consistent detection across contrasts and does not imply a causal or treatment-specific regulatory effect. Eleven candidate genes were selected for transcriptional validation from a broader sarcosine-relevant DMR-associated gene set, defined by the union of genes appearing in DMR Venn segments involving sKD comparisons (sKD vs. vSH and/or sKD vs. vKD) and pathway enrichment analyses. Selection was based on representation in sarcosine-relevant DMR comparisons, pathway enrichment, and established functional roles in epigenetic regulation, excitatory neurotransmission, or ECM remodeling (Figure 2D). The DMR context, methylation direction, and pathway assignment for each qPCR candidate are summarized in Supplementary Table S4.

### 2.3. Comparative Pathway Enrichment Signatures Associated with Epileptogenesis and Sarcosine Treatment

**Figure 3:**
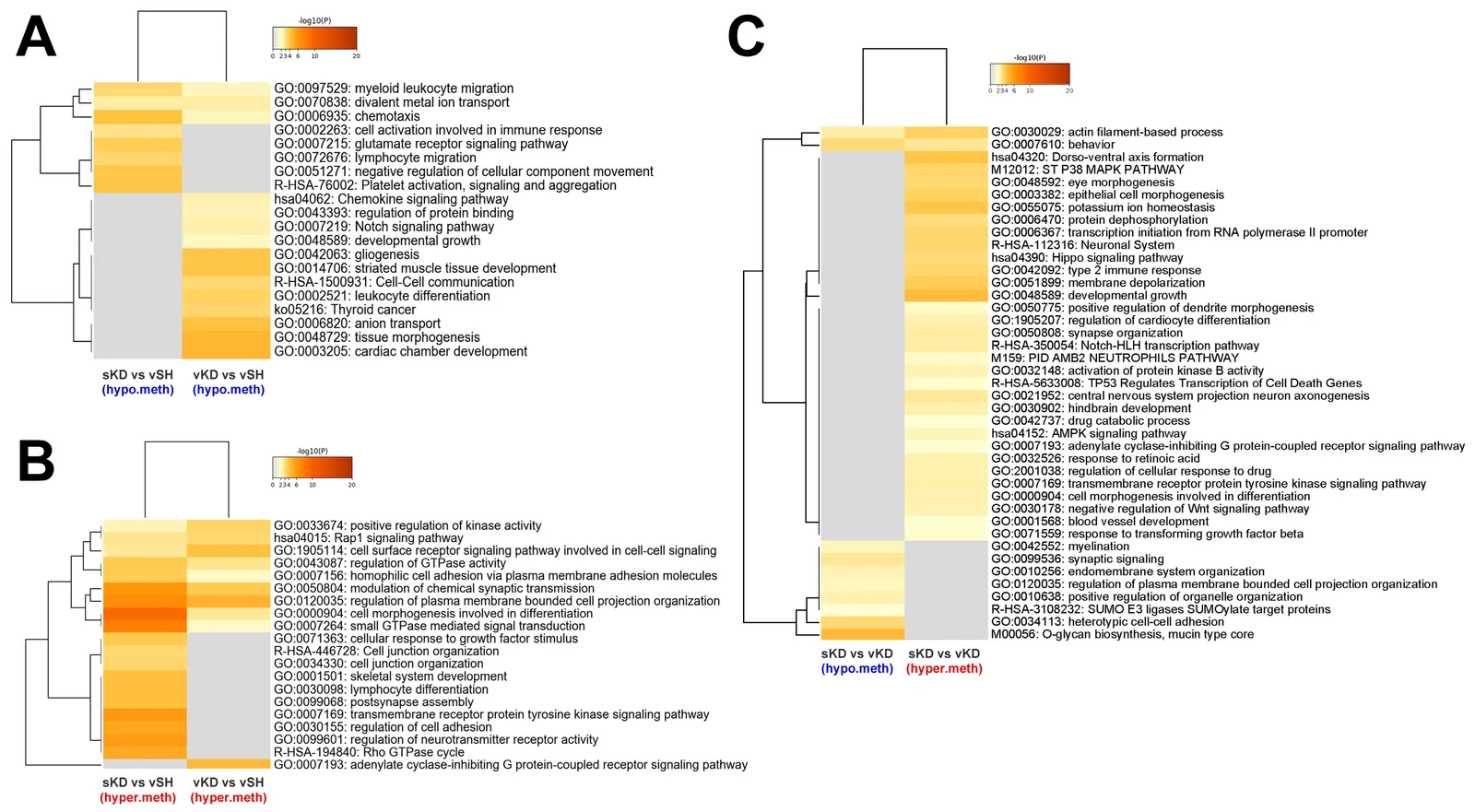
**Comparative enrichment profiles of DMR-associated genes.** Heatmaps show representative epiPathway/GO-enriched terms among hypomethylated (**A**) and hypermethylated (**B**) DMR-associated genes in the sKD vs. vSH and vKD vs. vSH comparisons, and among hypomethylated and hypermethylated DMR-associated genes in the sKD vs. vKD comparison (**C**). Color intensity represents −log10 (*p*-value); gray indicates no significant enrichment (*p* > 0.05). sKD, sarcosine-kindled; vKD, vehicle-kindled; vSH, vehicle-sham.

To investigate whether DMR-associated genes were overrepresented in coordinated biological processes relevant to epileptogenesis and sarcosine treatment, pathway enrichment analyses were performed using epiPathway and Gene Ontology (GO). Comparison of the vKD vs. vSH and sKD vs. vSH DMR-associated gene sets identified 217 significantly enriched pathways, including 13 shared epiPathway terms and 7 shared GO biological process categories. Representative enrichment heatmaps derived from these analyses across all three pairwise contrasts are shown in Figure 3. Among hypomethylated DMR-associated genes in the vKD vs. vSH and sKD vs. vSH comparisons, top enriched terms included myeloid leukocyte migration, chemotaxis, glutamate receptor signaling, Notch signaling, gliogenesis, and tissue morphogenesis (Figure 3A), indicating overrepresentation of annotations related to immune-cell migration, signaling, glial processes, and tissue morphogenesis in both kindled groups relative to sham. Among hypermethylated DMR-associated genes in the same comparisons, top enriched terms included positive regulation of kinase activity, modulation of chemical synaptic transmission, regulation of plasma membrane-bounded cell projection organization, postsynapse assembly, and regulation of neurotransmitter receptor activity (Figure 3B). In the direct treatment-related sKD vs. vKD contrast, hypermethylated DMR-associated genes were enriched for developmental growth, synapse organization, myelination, and TGF-β response, whereas hypomethylated DMR-associated genes were enriched for ECM organization, endomembrane system organization, and O-glycan biosynthesis (Figure 3C). These findings represent pathway-level enrichment among DMR-associated genes and should be interpreted as associations. Together, these analyses identify both shared and distinct methylation-associated pathway signatures across experimental contrasts, highlighting pathway-level associations involving synaptic signaling, neuroinflammatory responses, developmental programs, and extracellular matrix remodeling during kindling and with sarcosine treatment. These pathway signatures provided the framework for subsequent Metascape analyses focusing separately on kindling-associated and sarcosine-associated methylation programs.

### 2.4. Kindling-Associated Methylation Pathways Identified by Metascape Enrichment Analysis

**Figure 4:**
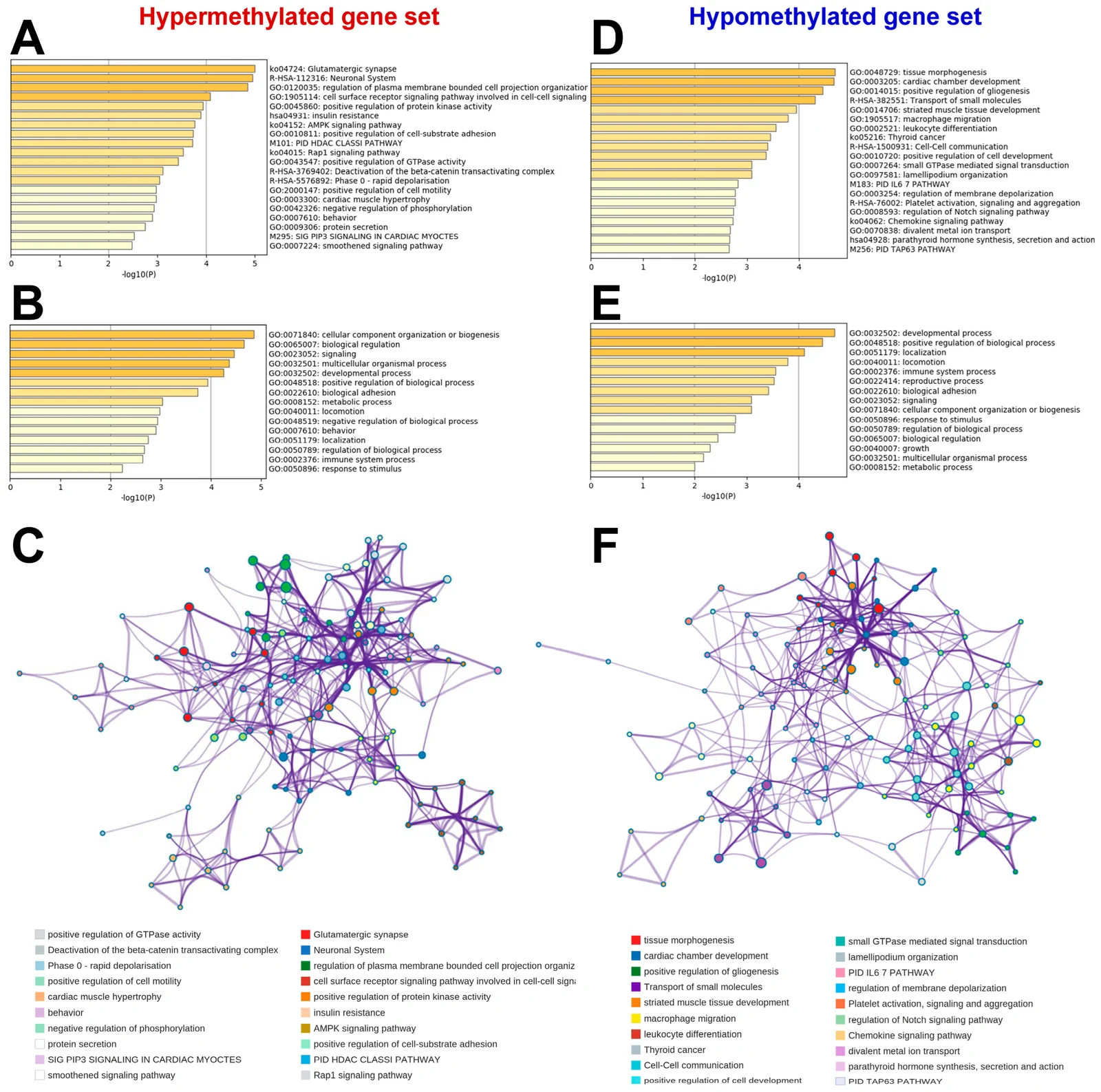
**Metascape enrichment analysis of DMR-associated genes in vehicle-kindled versus sham rats.** Enrichment analyses were performed separately for hypermethylated (**A**–**C**) and hypomethylated (**D**–**F**) DMR-associated genes in the vKD vs. vSH comparison. (**A**,**D**) Top enriched terms ranked by −log_10_ (*p*-value). (**B**,**E**) Parent Gene Ontology term enrichment. (**C**,**F**) Metascape pathway networks colored by cluster. vKD, vehicle-kindled; vSH, vehicle-sham.

To further characterize methylation-associated biological processes during kindling, Metascape enrichment analyses were performed separately on hypermethylated and hypomethylated DMR-associated genes identified in the vKD vs. vSH comparison (Figure 4). The hypermethylated gene set (n = 251) was significantly enriched for pathways related to glutamatergic synapse (ko04724), neuronal system (R-HSA-112316), and regulation of plasma membrane-bounded cell projection organization (GO:0120035) (Figure 4A–C). Additional enriched terms included regulation of neurotransmitter receptor activity, behavior, HDAC Class I pathway signaling, and neuronal depolarization-related processes. Pathway network analysis identified interconnected clusters containing annotations related to glutamatergic signaling, neuronal function, and cellular morphogenesis (Figure 4C).

The hypomethylated gene set (n = 284) was enriched for pathways related to IL-6 signaling, chemokine signaling, leukocyte differentiation, positive regulation of gliogenesis, regulation of membrane depolarization, small GTPase-mediated signaling, and lamellipodium organization (Figure 4D–F). Network analysis revealed a densely interconnected pathway architecture linking inflammatory signaling, developmental processes, and cellular remodeling pathways (Figure 4F). Collectively, these analyses indicate that kindling-associated DNA methylation differences were enriched near genes annotated to synaptic signaling, chromatin regulation, neuroinflammatory pathways, and structural remodeling networks.

### 2.5. Sarcosine-Associated Methylation Pathways Identified by Metascape Enrichment Analysis

**Figure 5:**
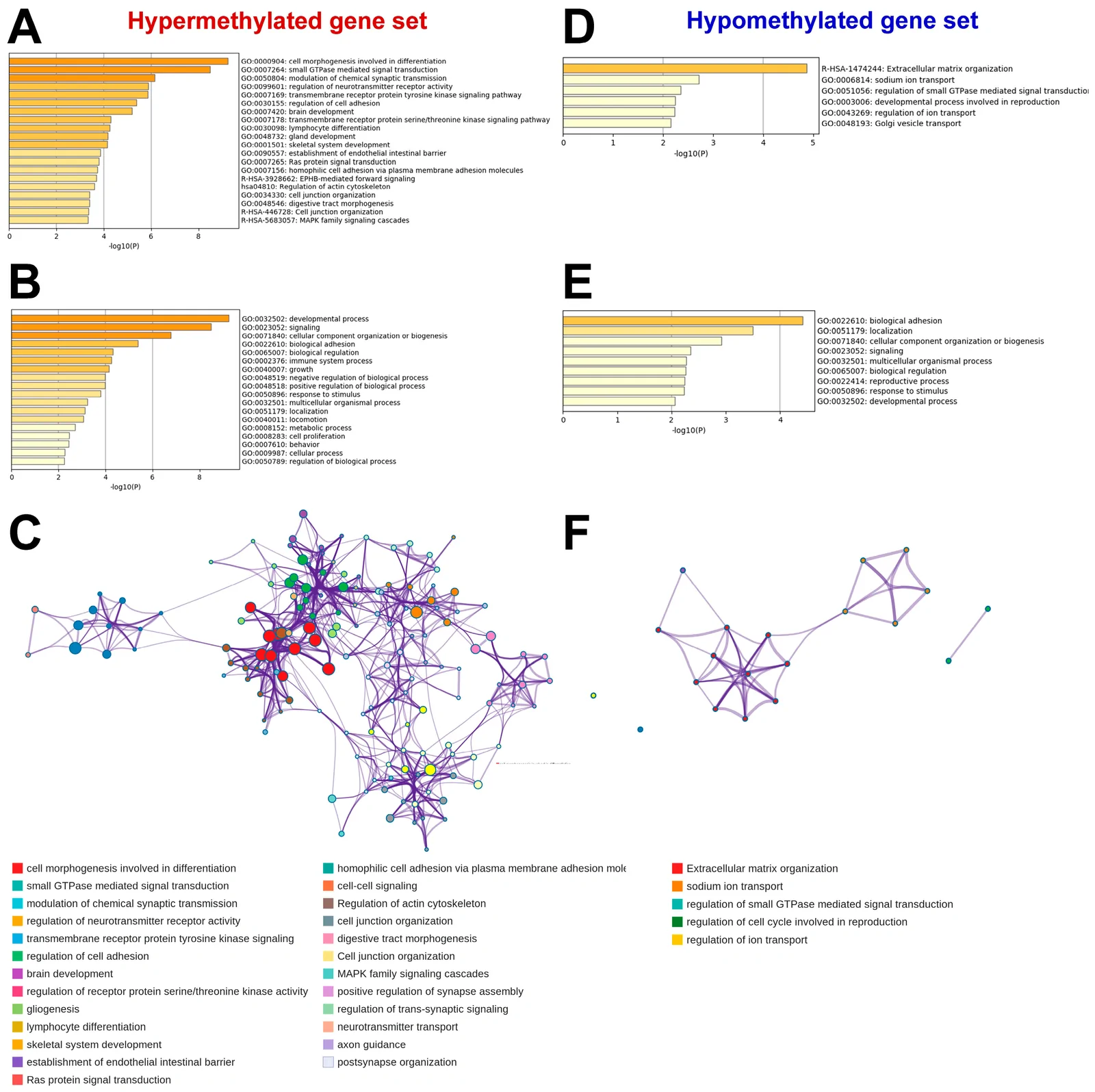
**Metascape enrichment analysis of DMR-associated genes in sarcosine-treated versus vehicle-treated kindled rats.** Enrichment analyses were performed separately for hypermethylated (**A–C**) and hypomethylated (**D–F**) DMR-associated genes in the sKD vs. vKD comparison. (**A**,**D**) Top enriched terms ranked by −log_10_(*p*-value). (**B**,**E**) Parent Gene Ontology term enrichment. (**C**,**F**) Metascape pathway networks colored by cluster. sKD, sarcosine-kindled; vKD, vehicle-kindled.

To characterize methylation-associated pathways linked to sarcosine treatment, Metascape enrichment analysis was performed on DMR-associated genes from the sKD vs. vKD comparison, which directly compares sarcosine- and vehicle-treated kindled rats, stratified by direction of methylation change (Figure 5).

The hypermethylated gene set (n = 295) was significantly enriched for developmental growth (GO:0048589), brain development (GO:0007420), chloride transmembrane transport (GO:1902476), response to transforming growth factor-β (GO:0071559), trans-synaptic signaling (GO:0099537), and positive regulation of synapse assembly (GO:0051965) (Figure 5A–C). Parent Gene Ontology (GO) analysis identified growth, developmental process, and negative regulation of biological process as the most enriched higher-level categories (Figure 5B). Network analysis revealed interconnected pathway clusters containing annotations related to developmental regulation, synaptic organization, and growth-related signaling (Figure 5C).

In contrast, the hypomethylated gene set (n = 85) was smaller and enriched for extracellular matrix organization (R-HSA-1474244), sodium ion transport (GO:0006814), regulation of small GTPase-mediated signal transduction (GO:0051056), and regulation of ion transport (GO:0043269) (Figure 5D–F). Parent GO analysis identified biological adhesion and localization as the most enriched higher-level categories (Figure 5E). Network analysis demonstrated two relatively discrete subnetworks containing annotations related to extracellular matrix organization and ion transport pathways (Figure 5F). Collectively, these findings indicate that sarcosine treatment was associated with a distinct pattern of DMRs in the kindled hippocampus, with DMR-associated genes enriched for annotations related to developmental regulation, synaptic organization, ion transport, and extracellular matrix remodeling.

### 2.6. Transcriptional Validation of Methylation-Associated Candidate Genes

**Figure 6:**
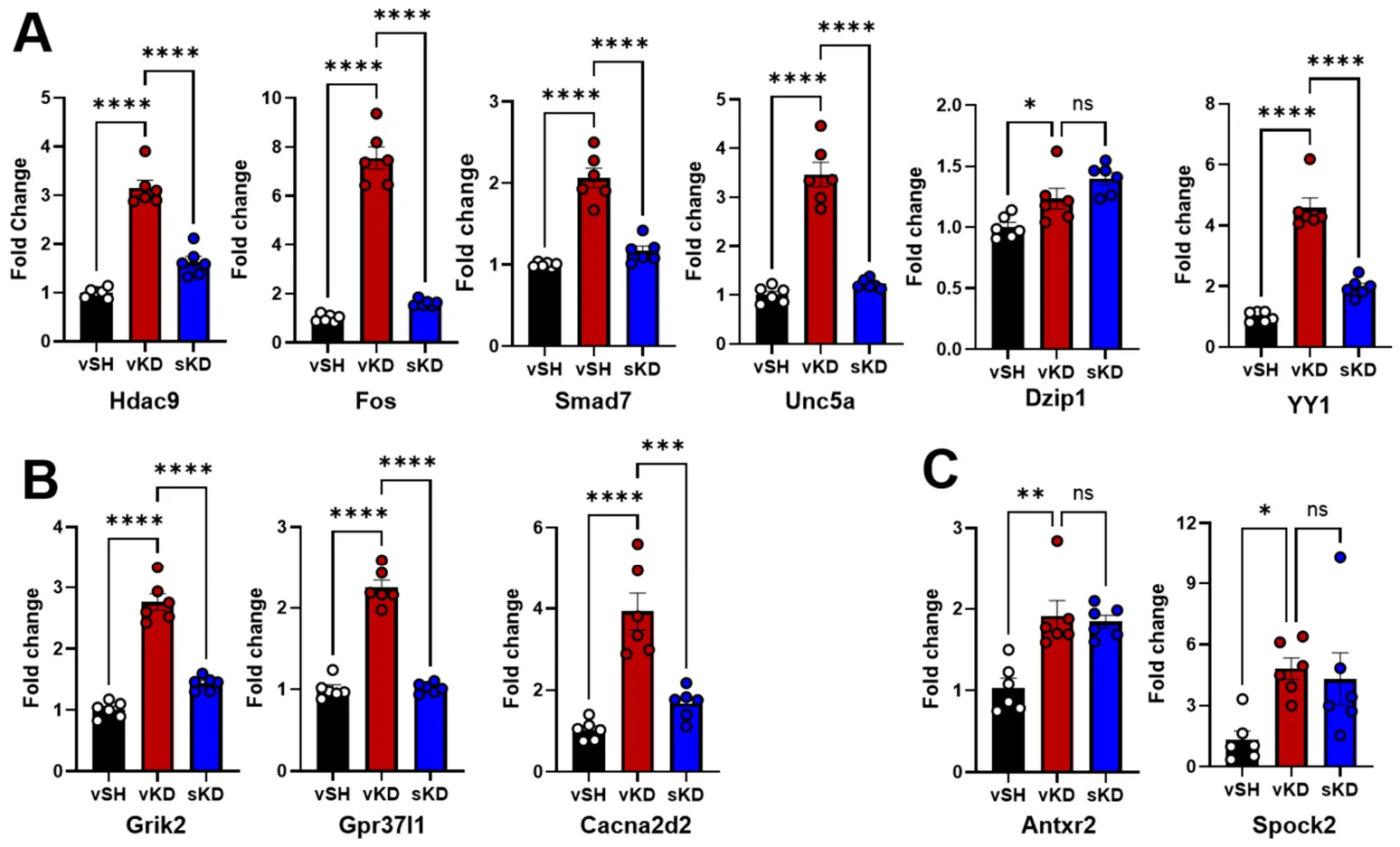
**Transcriptional validation of methylation-associated candidate genes.** Hippocampal mRNA expression of candidate genes in vehicle-sham (vSH), vehicle-kindled (vKD), and sarcosine-kindled (sKD) rats (n = 6 per group). (**A**) Transcriptional/epigenetic regulators. (**B**) Excitatory synaptic-signaling genes. (**C**) Extracellular-matrix-related genes. Data are mean ± SEM. One-way ANOVA with Tukey’s multiple-comparisons test was used. * *p* < 0.05, ** *p* < 0.01, *** *p* < 0.001, **** *p* < 0.0001; ns, not significant. Group-level values, ANOVA results, and Tukey-adjusted pairwise *p* values are provided in Supplementary Table S5.

To determine whether methylation-associated candidate genes showed group-dependent mRNA-expression differences during kindling, quantitative PCR was performed on 11 genes selected from the DMR-associated gene sets and pathway enrichment analyses. These candidates represented three functional categories: transcriptional and epigenetic regulation (Hdac9, Fos, Smad7, Unc5a, Dzip1, and Yy1), excitatory synaptic signaling (Grik2, Gpr37l1, and Cacna2d2), and extracellular matrix remodeling (Antxr2 and Spock2) (Figure 6). These genes were prioritized based on their representation in sarcosine-relevant DMR comparisons, enrichment analyses, and established biological relevance to epigenetic regulation, excitatory neurotransmission, and extracellular matrix remodeling.

All 11 genes exhibited significantly increased expression in vKD hippocampi compared with vSH controls, with group-level expression values and statistical results reported in Supplementary Table S5 (Figure 6). These differences indicate altered hippocampal mRNA abundance in vehicle-treated kindled rats. Sarcosine treatment during kindling (sKD) significantly reduced vKD-associated increases in mRNA expression of *Hdac9*, *Fos*, *Smad7*, *Unc5a*, *Grik2*, *Gpr37l1*, *Cacna2d2*, and *Yy1* relative to vKD. For these genes, sKD expression was not significantly different from vSH controls (Figure 6). In contrast, *Dzip1*, *Antxr2*, *and Spock2* remained significantly elevated in sKD relative to vSH and did not differ significantly from vKD, indicating no statistically significant attenuation by sarcosine under the tested conditions (Figure 6). Group-level qPCR expression values, variability estimates, omnibus ANOVA results, and Tukey-adjusted pairwise *p* values are provided in Supplementary Table S5.

Together, these findings demonstrate that genes selected from DMR-associated sets and enrichment analyses showed group-dependent mRNA-expression differences. The co-occurrence of DMR associations, pathway enrichment, and transcriptional differences identifies candidate molecular networks associated with kindling and sarcosine treatment.

## 3. Discussion

### 3.1. Mechanistic Insight into Kindling-Induced Epileptogenesis

In the present study, we combined genome-wide reduced representation bisulfite sequencing (RRBS), pathway enrichment analyses, and targeted transcriptional profiling to investigate DNA methylation-associated molecular networks during hippocampal kindling-induced epileptogenesis and their modulation by sarcosine. Consistent with our previous work, sarcosine significantly attenuated behavioral seizure progression in the rapid hippocampal kindling model under the present 5-day treatment protocol [19]. RRBS identified widespread group-associated DMRs, and qPCR identified altered hippocampal mRNA abundance in candidate genes annotated to transcriptional/epigenetic regulation, synaptic signaling, and extracellular matrix (ECM) remodeling. Because these analyses were performed in bulk hippocampal tissue, they cannot resolve the cell-type origins of the observed methylation or transcriptional differences.

Among the validated genes, several transcriptional and epigenetic regulators—including Hdac9, Fos, Smad7, Unc5a, and Yy1—were significantly upregulated in vKD relative to vSH and were reduced in sKD relative to vKD. HDAC9 has been implicated in chromatin regulation and neuronal plasticity [3,12,13]. In contrast, Fos is a canonical immediate-early gene that is induced by neuronal activity and seizures [4,10,21,22]. Smad7 encodes an inhibitory regulator of transforming growth factor-β (TGF-β) signaling. TGF-β-related pathways have been implicated in neuroinflammatory, blood–brain barrier, glial, and network-remodeling processes in experimental and human epilepsy [3,7,23,24]. However, the present data do not determine whether the observed Smad7 mRNA differences reflect altered TGF-β pathway activity or identify the cell type in which they occur.

In addition to transcriptional regulators, we identified altered mRNA expression of candidate genes annotated to excitatory synaptic signaling, including Grik2, Gpr37l1, and Cacna2d2. These findings are consistent with extensive evidence implicating glutamatergic dysfunction and aberrant synaptic plasticity in seizure development and progression [4,13,25,26]. The reductions in mRNA abundance observed in sKD relative to vKD were associated with sarcosine treatment; because sarcosine has multiple biological actions, the present design cannot determine the relative contributions of GlyT1 inhibition, one-carbon metabolism, or other downstream signaling effects [19,27]. Finally, Antxr2 and Spock2 exhibited kindling-associated transcriptional changes, but neither was significantly reduced by sarcosine relative to vKD under the conditions tested. The present findings therefore identify ECM-related genes as candidates associated with kindling but do not support transcriptional attenuation of these genes by sarcosine. ECM remodeling has been associated with structural plasticity, synaptic integrity, and epileptogenic network dysfunction in experimental and human epilepsy [3,28,29].

Collectively, these findings identify co-occurring DMR-associated pathway enrichments and candidate-gene mRNA differences involving transcriptional regulation, synaptic signaling, and ECM-related processes during rapid hippocampal kindling. Sarcosine was associated with attenuation of behavioral seizure progression and with reduced expression of eight candidate genes in this model. Because methylation assignment was based on nearest-TSS mapping, methylation and mRNA measurements were not directly correlated, and sarcosine has multiple biological actions, the study does not establish a methylation-dependent mechanism or causality for sarcosine’s effects.

### 3.2. Epigenetic Context of the qPCR-Validated Genes

RRBS analysis revealed group-associated DMRs across all experimental comparisons. The 11 genes selected for transcriptional validation were derived from a broader sarcosine-relevant DMR-associated gene set, defined by genes appearing in DMR Venn segments involving sKD comparisons (sKD vs. vSH and/or sKD vs. vKD) and pathway enrichment analyses. The candidates were prioritized based on representation in sarcosine-relevant DMR comparisons, pathway enrichment, and established functional roles in epigenetic regulation, excitatory neurotransmission, or ECM remodeling. Importantly, DMR-to-gene relationships were inferred using nearest-transcription-start-site mapping rather than direct methylation-expression correlation analyses. Therefore, the observed associations between methylation changes and gene expression should be interpreted as proximity-based candidate associations, rather than evidence of direct regulatory or causal relationships [3,12,14].

The combination of DMR mapping, pathway enrichment, and qPCR identifies these genes as candidates for further mechanistic investigation. Genes annotated to transcriptional regulation (*Hdac9*, *Fos*, *Smad7*, *Unc5a*, and *Yy1*), excitatory synaptic signaling (*Grik2*, *Gpr37l1*, and *Cacna2d2*), and extracellular matrix remodeling (*Antxr2* and *Spock2*) exhibited group-dependent mRNA-expression differences that occurred alongside methylation-associated pathway enrichments. Eight genes were reduced in sKD relative to vKD, whereas Dzip1, Antxr2, and Spock2 were not significantly altered by sarcosine treatment. Thus, the findings are consistent with sarcosine-associated changes across multiple candidate molecular programs, but they do not establish that GlyT1 inhibition or a specific DMR caused the observed transcriptional differences.

### 3.3. Kindling-Associated Methylation Pathways

Metascape analyses demonstrated that DMR-associated genes in the vKD versus vSH comparison showed distinct enrichment profiles when stratified by the direction of methylation change. Hypermethylated DMR-associated genes were enriched for glutamatergic synapse, neuronal system, neurotransmitter receptor activity, and HDAC-related pathways, whereas hypomethylated genes were enriched for chemokine signaling, leukocyte differentiation, gliogenesis, and structural remodeling processes. These findings are consistent with previous reports of DNA methylation differences associated with neuronal signaling and inflammatory pathways in experimental epileptogenesis [10,12,13,14].

The enrichment of glutamatergic and broader neuronal system pathways within the hypermethylated gene set is consistent with the established involvement of excitatory synaptic signaling and network plasticity in kindling. Dysregulation of glutamatergic signaling is a well-established driver of seizure initiation and propagation, and abnormal plasticity within excitatory networks is thought to contribute to the progressive lowering of seizure threshold during kindling. However, pathway enrichment does not establish that these pathways were activated or that the identified DMRs altered glutamatergic gene expression. The occurrence of glutamatergic pathway enrichment alongside increased mRNA expression of selected excitatory-signaling genes is compatible with the context-dependent relationship between DNA methylation and transcription. DNA methylation effects can differ according to genomic location, chromatin context, cell type, and the regulatory elements involved. Similar complexity has been reported in experimental and human epilepsy studies [3,12,14].

The enrichment of HDAC-related pathways is of interest given the increased Hdac9 mRNA expression observed in kindled animals. Histone deacetylases are increasingly recognized as important regulators of activity-dependent gene expression and neuronal plasticity, and dysregulated HDAC signaling has been implicated in several neurological disorders, including epilepsy [3,13,30]. The present data do not determine whether the HDAC-related enrichment and Hdac9 mRNA difference reflect a shared regulatory mechanism; nevertheless, they identify chromatin-related processes as candidates for further investigation. In contrast, hypomethylated DMR-associated genes were enriched for pathways related to chemokine signaling, leukocyte differentiation, gliogenesis, and structural remodeling. This enrichment profile is consistent with literature implicating neuroimmune and glial processes in seizure-associated network remodeling. Neuroinflammatory responses have been associated with seizure susceptibility, gliosis, and longer-term circuit remodeling in experimental epilepsy [1,3,13,31]. Because the analyses used bulk hippocampal tissue, the observed enrichment cannot be assigned to specific immune, glial, neuronal, or vascular cell populations.

Together, these results indicate that kindling-associated DMRs were assigned to genes enriched for annotations related to excitatory synaptic signaling, chromatin regulatory mechanisms, neuroinflammatory pathways, and structural plasticity. These results generate hypotheses concerning molecular networks associated with rapid hippocampal kindling but do not establish a bidirectional epigenetic program, direct effects on transcription, or a causal role in the transition to epilepsy.

### 3.4. Sarcosine-Associated Epigenetic Remodeling

The direct comparison between sarcosine-treated and vehicle-treated kindled rats identified distinct methylation-associated pathway enrichments. Hypermethylated DMR-associated genes were enriched for developmental growth, brain development, chloride transmembrane transport, synaptic organization, and TGF-β-responsive pathways, whereas hypomethylated DMR-associated genes were enriched for ECM organization, ion transport, and small GTPase-mediated signaling.

Developmental growth and brain-development annotations may be relevant to seizure-associated structural plasticity, including synaptic reorganization and axonal remodeling. However, the present data do not show that sarcosine reverses maladaptive developmental programs or directly affects structural remodeling [32,33]. Likewise, the enrichment of chloride transmembrane transport annotations highlights a pathway relevant to inhibitory signaling and network excitability but does not demonstrate altered chloride homeostasis or direct regulation of KCC2, NKCC1, or other chloride transporters [34,35].

TGF-β-responsive pathway enrichment occurred alongside reduced Smad7 mRNA expression in sKD relative to vKD. However, these observations do not establish altered TGF-β pathway activity, a direct methylation–expression relationship, or a contribution to sarcosine-associated seizure attenuation. TGF-β signaling has been implicated in neuroinflammatory, blood–brain barrier, and epileptogenic processes [3,7].

Hypomethylated DMR-associated genes were also enriched for ECM organization. Although Antxr2 and Spock2 displayed kindling-associated mRNA differences, neither gene was significantly reduced by sarcosine relative to vKD. Therefore, the present data do not support sarcosine-mediated transcriptional attenuation of these ECM candidates. ECM and perineuronal-net remodeling have been associated with synaptic plasticity and epileptogenic network dysfunction, but PNNs and ECM structure were not directly examined here [25,36,37,38].

The sKD-versus-vKD pattern was not simply the inverse of the vKD-versus-vSH pattern, indicating that sarcosine-associated DMRs did not uniformly return toward the sham-associated profile. Sarcosine has multiple relevant biological actions, including GlyT1 inhibition and effects on one-carbon metabolism; therefore, this study cannot determine the mechanism responsible for the observed DMR pattern [19]. Overall, sarcosine treatment was associated with a distinct hippocampal DMR profile during rapid kindling. Future studies incorporating selective GlyT1 perturbation, metabolic controls, cell-type-resolved analyses, and functional measurements of candidate pathways are needed to define its mechanism.

### 3.5. Epigenetic Impact of Interventions Targeting Epileptogenesis

A growing body of evidence indicates that epigenetic mechanisms are associated with epileptogenesis and may represent therapeutic targets [3,11,13]. Several antiseizure drugs and metabolic interventions, including valproic acid and ketogenic dietary approaches, have been reported to influence DNA methylation, histone modification, and chromatin-associated signaling pathways [16,39]. However, relatively few studies have examined genome-wide methylation patterns during epileptogenesis in the context of an intervention while also assessing candidate-gene transcriptional changes.

The present study identifies methylation-associated and transcriptional differences accompanying sarcosine treatment during rapid hippocampal kindling. These observations do not establish that epigenetic modulation mediates sarcosine-associated attenuation of behavioral seizure progression. Recent pharmacological studies of GlyT1 inhibition support further investigation of glycine transport as a modifier of seizure susceptibility [40]. Because sarcosine can affect GlyT1 activity, one-carbon metabolism, and downstream neurotransmitter signaling, its mechanism cannot be inferred from the present design.

### 3.6. Clinical Implications and Future Directions

The present results identify candidate methylation-associated molecular networks for mechanistic study in rapid hippocampal kindling; they do not establish clinical efficacy or disease modification. Conventional antiseizure medications primarily control seizures, while interventions intended to prevent or alter epileptogenesis require evidence that they modify disease development rather than acute seizure expression [2,3]. Accordingly, whether sarcosine or GlyT1 inhibition can prevent, halt, or reverse epilepsy remains unknown.

Future studies should use cell-type-resolved methylome and transcriptome profiling, longitudinal sampling, and integrated electrophysiological, molecular, and behavioral outcomes. Experiments using selective GlyT1 inhibitors or genetic manipulation, alongside controls for one-carbon metabolic effects, will be necessary to distinguish possible mechanisms of sarcosine action. The rapid hippocampal kindling model, the 5-day sarcosine pretreatment regimen, the single tested dose, and behavioral seizure scoring limit extrapolation to spontaneous recurrent-seizure models, other etiologies, females, and clinical epilepsy. Locus-specific epigenetic editing may offer a future strategy to test whether individual regulatory regions associated with candidates such as Hdac9, Fos, or Smad7 affect seizure susceptibility or kindling progression. Such experiments should first establish cell type, locus-specific methylation status, transcriptional consequence, and functional relevance before therapeutic implications are considered.

### 3.7. Study Limitations

Several limitations should be considered. First, DMR-associated genes were assigned by nearest-transcription-start-site mapping rather than direct methylation–expression correlation analysis. Thus, DMR-to-gene relationships should be interpreted as proximity-based associations rather than evidence of direct regulatory or causal interactions. Second, RRBS and qPCR were performed in bulk hippocampal tissue; therefore, the observed methylation and transcriptional differences cannot be assigned to specific neuronal, glial, vascular, or immune-cell populations. Cell-type-resolved methylome and transcriptome analyses would help define the cellular sources of these associations. Third, transcriptional validation was limited to targeted mRNA measurements and did not assess protein expression. In addition, RRBS samples CpG-dense regions of the methylome and may not detect all relevant methylation differences. Finally, sarcosine was administered at a single dose (3 g/kg, i.p.) during the 5-day rapid hippocampal kindling protocol. Thus, the study cannot determine dose dependence, distinguish the contributions of GlyT1 inhibition and one-carbon metabolic effects, or establish whether the findings generalize to other epilepsy models or spontaneous recurrent seizures. Although electrographic activity was recorded during stimulation sessions, quantitative afterdischarge measures, including threshold and duration, were not analyzed. Therefore, the present assessment of kindling progression is based primarily on behavioral Racine scoring, and the findings should not be interpreted as demonstrating corresponding changes in electrographic seizure susceptibility or afterdischarge characteristics.

## 4. Materials and Methods

### 4.1. Ethical Approval and Animal Care

All animal procedures were conducted in an Association for Assessment and Accreditation of Laboratory Animal Care International (AAALAC)-accredited facility and were approved by the Institutional Animal Care and Use Committee of the Legacy Research Institute (protocol number: 114-2020). Male Sprague–Dawley rats (280–300 g) were obtained from Jackson Laboratory (Sacramento, CA, USA). Animals were acclimated for one week before experimentation and housed in pairs in standard cages under controlled temperature and humidity conditions on a 12 h light/dark cycle (lights on at 07:00 h) with *ad libitum* access to food and water throughout the study. Following electrode-implantation surgery, rats received a single subcutaneous dose of meloxicam (1 mg/kg) for postoperative analgesia. Animals were monitored daily for general health, body condition, and signs of postoperative discomfort or distress. All procedures were performed in accordance with the approved IACUC protocol and with measures to minimize pain, suffering, and distress.

### 4.2. Rat Kindling and Treatment

The hippocampal kindling model was performed as previously described [19]. Eight-week-old male Sprague–Dawley rats were surgically implanted with bipolar electrodes (Plastics One, Roanoke, VA, USA) targeting the left dorsal hippocampus (coordinates relative to bregma: AP −5.0 mm, ML +5.0 mm, DV −7.5 mm) according to the Rat Brain Atlas of Paxinos and Watson, sixth edition [41], under 1–3% inhaled isoflurane anesthesia; the electrodes were secured with dental acrylic and skull screws. Following a 10-day postoperative recovery period, animals underwent a rapid hippocampal kindling protocol over five consecutive days [19]. Rats were randomly assigned to one of three experimental groups (n = 6 per group): (i) vehicle-sham (vSH), which received sham stimulation and vehicle treatment; (ii) vehicle-kindled (vKD), which received vehicle treatment during the kindling protocol; and (iii) sarcosine-kindled (sKD), which received sarcosine (3 g/kg, i.p.; Cat. No. 131776, Sigma-Millipore, Burlington, MA, USA) during the kindling protocol. Sarcosine or vehicle was administered intraperitoneally 30 min before each daily stimulation session. One sarcosine dose and pretreatment interval were evaluated; dose–response effects and alternative treatment schedules were not assessed.

Each kindling session consisted of six electrical stimulations delivered using a Grass S-88 stimulator (A-M Systems, Sequim, WA, USA) (1 ms biphasic square-wave pulses, 200 μA, 50 Hz, 10 s duration) separated by 30 min intervals. Behavioral seizure severity was scored according to the Racine scale [19,42], and electrographic seizure activity was monitored using the PowerLab data acquisition system (ADInstruments, Colorado Springs, CO, USA) as previously described [19]. During stimulation and recording sessions, rats were freely moving in individual recording chambers and returned to their home cages after each session. Behavioral Racine scoring was the primary outcome used to assess kindling progression in the present study. The vSH group served as the baseline control for comparisons of behavioral seizure progression and gene expression.

### 4.3. Tissue Collection

Hippocampal tissue from sarcosine-treated (sKD) and vehicle-treated (vKD) kindled rats was collected 24 h after the final stimulation session. Age-matched vehicle-treated sham animals (vSH) served as baseline controls. Rats were deeply anesthetized with isoflurane and euthanized by rapid decapitation. The hippocampus was rapidly dissected, with tissue from the right hemisphere allocated for DNA extraction and reduced representation bisulfite sequencing (RRBS), and tissue from the left hemisphere used for RNA isolation and quantitative real-time PCR (qPCR) analyses.

### 4.4. Reduced Representation Bisulfite Sequencing (RRBS)

Genomic DNA was extracted from hippocampal tissue using Qiagen protocols by the Gene Profiling Shared Resource Core at Oregon Health & Science University (OHSU, Portland, OR, USA). DNA concentration and quality were assessed by UV spectrophotometry and fragment analysis using a TapeStation system (Agilent Technologies, Inc., Santa Clara, CA, USA). RRBS library preparation and methylation profiling were performed by the KCVI Epigenetics Core at OHSU, and next-generation sequencing was conducted by the OHSU Massively Parallel Sequencing Shared Resource Core.

RRBS libraries were generated from 200 ng of genomic DNA per sample. Six animals were initially included in each experimental group; however, one sKD sample failed RRBS quality control and was excluded from methylation analyses. Consequently, RRBS was performed using n = 6 for vSH, n = 6 for vKD, and n = 5 for sKD, following established protocols [43]. Genomic DNA was digested overnight with MspI (New England Biolabs, Ipswich, MA, USA), and libraries were prepared using the NEXTflex Bisulfite-Seq Kit (Bioo Scientific, Austin, TX, USA). Bisulfite conversion was performed using the EZ DNA Methylation-Gold Kit (Zymo Research, Irvine, CA, USA), followed by PCR amplification using NEBNext Multiplex Oligos (New England Biolabs). Libraries were sequenced on an Illumina NextSeq 500 platform using a 75 bp paired-end, high-output protocol (Illumina, San Diego, CA, USA), generating approximately 40 million reads per sample.

Library quality and bisulfite conversion efficiency were assessed prior to downstream analyses. Bisulfite conversion efficiency exceeded 99% in all samples. Mean CpG coverage depth was approximately 10×, with each sample achieving ≥10× coverage at 716,512–1,337,815 CpG sites. Alignment rates to the Rnor_6.0 reference genome ranged from 70.5% to 75.1% (mean 72.9%), indicating high-quality sequencing data suitable for differential methylation analysis. Although this coverage threshold supports reliable estimation at retained CpGs, the relatively modest sequencing depth may have reduced sensitivity to detect subtle methylation differences and excluded low-coverage loci from analysis.

### 4.5. Differential Methylation Analysis and Pathway Enrichment

Differentially methylated cytosines (DMCs) and differentially methylated regions (DMRs) were identified using established bioinformatic pipelines [43]. DMC analysis was performed using the Limma package (v3.34.9). Only CpG sites with a minimum coverage of 10× in at least three biological replicates per group were retained for analysis, yielding a total of 644,003 CpGs. Sequencing reads were aligned to the *Rattus norvegicus* reference genome assembly (Rnor_6.0) using Ensembl gene annotations (release 80). Methylation values were arcsine-transformed prior to statistical analysis to improve distributional normality. DMRs were identified using the Comb-p algorithm. Regions containing at least two adjacent CpGs with FDR-corrected *p* values < 0.05 and located within 300 bp of one another were defined as DMRs. Hypermethylated and hypomethylated DMRs were classified using a methylation-difference threshold of ≥10% or ≤−10%, respectively.

To investigate the biological significance of DMR-associated genes, functional enrichment analyses were performed using epiPathway and Gene Ontology (GO) to identify enriched molecular pathways and biological processes across experimental comparisons. Comparative enrichment profiles derived from these analyses are presented in Figure 3. Metascape (accessed in 2023; metascape.org) was subsequently used for pathway enrichment and network analyses of hypermethylated and hypomethylated DMR-associated gene sets in the vKD vs. vSH and sKD vs. vKD comparisons (Figure 4 and Figure 5). These complementary analyses were used to identify molecular processes and pathway-level associations related to rapid hippocampal kindling and sarcosine treatment.

Complete DMR lists for all comparison groups are provided in Supplementary Tables S1–S3. DMRs were assigned to their nearest genes using gene-centric mapping based on the nearest transcription start site (TSS). Candidate genes selected for transcriptional validation were identified from these DMR-associated gene lists and pathway enrichment analyses. Associations between DNA methylation changes and gene expression were therefore inferred through DMR-to-gene mapping and functional pathway relationships rather than direct sample-wise methylation–expression correlation analyses. Because proximity-based DMR annotation does not establish regulatory interactions, DMR-associated genes are referred to throughout as methylation-associated candidates rather than direct methylation-regulated targets. A summary of available DMR annotations, methylation directions, and functional categories for the 11 genes selected for qPCR validation is provided in Supplementary Table S4.

### 4.6. Quantitative Real-Time PCR (qPCR)

Total RNA was extracted from hippocampal tissue using the RNeasy Mini Kit (Cat. No. 74104, Qiagen, Germantown, MD, USA). RNA concentration and purity were assessed using a NanoDrop ND-1000 spectrophotometer (Thermo Fisher Scientific, Waltham, MA, USA). First-strand cDNA was synthesized using the Promega Reverse Transcription System (Cat. No. A3500, Promega, Leiden, The Netherlands) in a 20 μL reaction according to the manufacturer’s instructions (42 °C for 40 min, followed by 85 °C for 5 min). cDNA was stored at 4 °C until qPCR analysis.

Quantitative PCR was performed using SYBR Green Universal Master Mix (Cat. No. 4309155, Thermo Fisher Scientific) and gene-specific primers targeting extracellular matrix remodeling (Antxr2, Spock2), excitatory synaptic signaling (Grik2, Gpr37l1, and Cacna2d2), and transcriptional/epigenetic regulation (Hdac9, Fos, Smad7, Unc5a, Dzip1, and Yy1) (Table 1). Gapdh served as the endogenous reference gene. Each 20 μL reaction contained 10 μL SYBR Green Master Mix, 0.8 μM each forward and reverse primer, and 5 μL cDNA template. No-template controls were included in each run to monitor reagent contamination. Each biological sample was analyzed in technical triplicate, and the mean Ct value of the technical replicates was used for downstream analysis.

Reactions were run on a QuantStudio™ 5 Real-Time PCR System (Applied Biosystems, Thermo Fisher Scientific, Waltham, MA, USA) using the following conditions: 95 °C for 5 min, followed by 40 cycles of 95 °C for 10 s and 60 °C for 30 s. Relative gene expression was calculated using the 2^−ΔΔCt^ method, normalized to *Gapdh*, with the vSH group used as the calibrator group and set to 1.

### 4.7. Statistical Analysis

Relative gene expression was calculated using the 2^−ΔΔCt^ method. Expression data were normalized to Gapdh, whose stability was verified using the geNorm and NormFinder algorithms (GenEx, MultiD).

Data are presented as mean ± SEM. Normality was assessed prior to statistical testing. For comparisons between two groups, Student’s *t*-test or the Mann–Whitney U test was used, as appropriate. For comparisons among multiple groups, one-way or two-way ANOVA, followed by Tukey’s multiple-comparisons test, was performed as appropriate. For each qPCR target gene, expression among the vSH, vKD, and sKD groups was analyzed using one-way ANOVA followed by Tukey’s multiple-comparisons test. Statistical significance was defined as *p* < 0.05. All analyses were conducted using GraphPad Prism 10 (GraphPad Software, San Diego, CA, USA). Group means ± SEM, sample sizes, omnibus ANOVA results, and Tukey-adjusted pairwise *p* values for qPCR analyses are provided in Supplementary Table S5.

## 5. Conclusions

Using rapid hippocampal kindling, RRBS identified group-associated hippocampal DMR patterns in vehicle-kindled, sarcosine-kindled, and sham rats. DMR-associated genes were enriched for annotations related to transcriptional regulation, synaptic signaling, and extracellular matrix remodeling. Targeted qPCR showed increased mRNA expression of 11 selected candidate genes in vehicle-kindled hippocampi; sarcosine treatment was associated with reduced expression of eight candidates and attenuation of behavioral seizure progression under the tested regimen.

These findings identify methylation-associated molecular networks accompanying rapid hippocampal kindling and sarcosine treatment. They do not demonstrate that individual DMRs regulate gene expression or that methylation changes mediate sarcosine-associated seizure attenuation. Because sarcosine has multiple biological actions and GlyT1 dependence was not directly tested, further cell-type-resolved and mechanistic studies are needed to determine whether the identified pathways contribute to epileptogenesis or represent targets for disease-modifying intervention.

## Figures and Tables

**Table 1:** Primer sequences used for quantitative real-time PCR.

Gene	Forward Primer (5′-3′)	Reverse Primer (5′-3′)
*Antxr2*	CGCCAATTAAGGGTCGTCTG	CGGGAGAAGTTTATGCACCG
*Cacna2d2*	GCGAGGTAAACAACGAGGAC	CACTGAAGAACCTGCCAACC
*Dzip1*	TTCCACCCGAAGAAGTACCC	TTTCTTGAGTTTTGGGGCGG
*Fos*	TGCAAGATCCCCAATGACCT	TGAGAAGAGGCAGGGTGAAG
*Gpr37l1*	AGCAGTGTGAGAGTCAGCTT	TGTTGCAGACGTTTTCAGGG
*Grik2*	ACTCAGGTTTGCTGGATGGA	CAGGGTTTGTGTCGATTGCA
*Hdac9*	TGTGAATACGAATGCAGCCG	CTTGCAACAGCCACCATCTT
*Smad7*	GGCTTCACCGTGCAGATTAG	TGAAGATGACCTCCAGCCAG
*Spock2*	ACTGTGATGACATCGTGGGT	CCAGATGTAGCCTCCGTCAT
*Unc5a*	AAGAAGGAAGGGCTGGACTC	CTGGTAGGTGGTAGTGGTGG
*Yy1*	GCAAGAAGAGTTACCTGGGC	ACCTGCTTCTGTTCCCACTT
*Gapdh*	GGATACTGAGAGCAAGAGAGA	TTATGGGGTCTGGGATGGAA

## Data Availability

The processed differentially methylated region (DMR) datasets underlying the analyses reported in this study are provided in Supplementary Tables S1–S3 for the vKD vs. vSH, sKD vs. vSH, and sKD vs. vKD comparisons, respectively. These tables include DMR genomic coordinates, methylation direction, and associated gene annotations. The raw RRBS sequencing files from this study are not available for public deposition. All other data supporting the findings of this study are included in the article and its Supplementary Materials. Further inquiries can be directed to the corresponding author.

## Supplementary Materials

The following supporting information can be downloaded at: https://www.mdpi.com/article/10.3390/ijms27177938/s1.
